# Carbapenem-resistant *Klebsiella pneumoniae* among hospitalized patients in Cape Town, South Africa: molecular epidemiology and characterization

**DOI:** 10.1093/jacamr/dlae050

**Published:** 2024-03-25

**Authors:** Gert Marais, Clinton Moodley, Shantelle Claassen-Weitz, Fadheela Patel, Elizabeth Prentice, Hafsah Tootla, Nyasha Nyakutira, Katie Lennard, Kessendri Reddy, Colleen Bamford, Abraham Niehaus, Andrew Whitelaw, Adrian Brink, Cape Town Antimicrobial Stewardship Study Alliance, Claudine Page, Elizabeth Schoeman, Elizma de Klerk, Karin Lategan, Karlien Pienaar, Liezl Henning, Mandy Du Plessis, Nomfundo Maseko, Salome Nel, Melenie Narainsamy, Michelle Vermeulen, Narissa du Toit, Teresa van Heerden, Liza Sitharam, Asa Barendse, Dane Nagel, Jacqueline Prince, Letitia Vass, Rileen Strauss, Rushana Fakier, Catherine Samuel, Marelieze van Zyl, Leigh-Ann Isaacs, Shareefa Hendricks, Amy Dodd, Reecka Daniels, Widaad Zemanay, Judi Van Heerden, Nchimunya Hapeela, Parveen Brown, Zubayr Daniels, Sharon Vasuthevan, Enid Scott, Esmeralda Ricks, Patricia Curle, Justyna Wojno

**Affiliations:** Division of Medical Microbiology, University of Cape Town, Cape Town, Western Cape, South Africa; National Health Laboratory Service Laboratory, Groote Schuur Hospital, Cape Town, Western Cape, South Africa; Division of Medical Microbiology, University of Cape Town, Cape Town, Western Cape, South Africa; National Health Laboratory Service Laboratory, Groote Schuur Hospital, Cape Town, Western Cape, South Africa; Division of Medical Microbiology, University of Cape Town, Cape Town, Western Cape, South Africa; Division of Medical Microbiology, University of Cape Town, Cape Town, Western Cape, South Africa; Division of Medical Microbiology, University of Cape Town, Cape Town, Western Cape, South Africa; National Health Laboratory Service Laboratory, Groote Schuur Hospital, Cape Town, Western Cape, South Africa; Division of Medical Microbiology, University of Cape Town, Cape Town, Western Cape, South Africa; Medical Microbiology, National Health Laboratory Service, Red Cross War Memorial Children’s Hospital, Cape Town, South Africa; Division of Medical Microbiology, University of Cape Town, Cape Town, Western Cape, South Africa; Division of Computational Biology, University of Cape Town, Cape Town, Western Cape, South Africa; Division of Medical Microbiology, Stellenbosch University, Cape Town, Western Cape, South Africa; National Health Laboratory Service, Tygerberg Hospital, Cape Town, Western Cape, South Africa; Division of Medical Microbiology, University of Cape Town, Cape Town, Western Cape, South Africa; Division of Medical Microbiology, Pathcare, Cape Town, South Africa; Division of Medical Microbiology, Ampath, Cape Town, South Africa; Division of Medical Microbiology, Stellenbosch University, Cape Town, Western Cape, South Africa; National Health Laboratory Service, Tygerberg Hospital, Cape Town, Western Cape, South Africa; Division of Medical Microbiology, University of Cape Town, Cape Town, Western Cape, South Africa; National Health Laboratory Service Laboratory, Groote Schuur Hospital, Cape Town, Western Cape, South Africa; Institute of Infectious Disease and Molecular Medicine, University of Cape Town, Cape Town, Western Cape, South Africa

## Abstract

**Background:**

The molecular epidemiology of carbapenem-resistant Enterobacterales in Cape Town remains largely unknown.

**Objectives:**

This study aimed to describe the molecular epidemiology, resistome, virulome and mobilome of carbapenem-resistant *Klebsiella pneumoniae* (CRKP) within Cape Town to guide therapy, antimicrobial stewardship and infection prevention and control practices.

**Methods:**

Eighty-five CRKP isolates from hospitalized patients underwent WGS as part of a prospective, multicentre, cross-sectional study, conducted between 1 November 2020 and 30 November 2022, across public-sector and private-sector hospitals in Cape Town, South Africa.

**Results:**

MLST revealed three novel types, ST6785, ST6786 and ST6787, while the most common were ST219, ST307, ST17, ST13 and ST2497. Different predominant clones were noted in each hospital. The most common carbapenemase gene was *bla*_OXA-48-like_, detected in 71% of isolates, with *bla*_NDM_ detected in 5%. Notably, co-detection of two carbapenemase genes (*bla*_OXA-48-like_ and *bla*_NDM_) occurred in 13% of isolates. The yersiniabactin siderophore was detected in 73% of isolates, and was most commonly associated with the ICE*Kp*5 mobile element. All carbapenemases were located on plasmids. The genes *bla*_OXA-181_ and *bla*_OXA-232_ colocalized with a ColKP3 replicon type on assembled contigs in 83% and 100% of cases, respectively.

**Conclusions:**

CRKP epidemiology in Cape Town reflects institutionally dominant, rather than regional, clones. The most prevalent carbapenemase gene was *bla*_OXA-48-like_, in keeping with CRKP epidemiology in South Africa in general. Emerging clones harbouring both *bla*_OXA-48-like_ and *bla*_NDM_, such as ST17, ST2497 and the novel ST6787, are a concern due to the limited availability of appropriate antimicrobial agents in South Africa.

## Introduction

The burden of difficult-to-treat MDR Gram-negative bacteria, including carbapenem-resistant Enterobacterales (CRE), is rapidly increasing in South Africa. Locally, institutional epidemiology of carbapenemase-producing Enterobacterales (CPE) is diverse and constantly evolving.^1–4^ While new β-lactam/β-lactamase inhibitor combinations (BLICs) are becoming more accessible, the associated cost and carbapenemase-enzyme specific activity requires local molecular epidemiology data to inform rational, cost-effective roll-out.^1^

The first carbapenemase enzymes detected in clinical isolates from South Africa were NDM and *Klebsiella pneumoniae* carbapenemase (KPC) in 2011 from the Gauteng province.^3,5^ Subsequently, carbapenem-hydrolysing OXA-48, and its variant OXA-181, were detected in 2012.^6^ More recently, a large clonal outbreak of OXA-181 in *K. pneumoniae* ST307 occurred across multiple provinces in the North-Eastern and North-Western regions of South Africa and a smaller outbreak of OXA-181-producing *K. pneumoniae* was reported from a public sector haematology/oncology unit in Cape Town.^7,8^

The carbapenemases IMI,^9^ VIM,^3,10,11^ GES,^3,11,12^ KPC^3^ and IMP^10,11^ have been infrequently documented in South Africa, with OXA-48 and its variants, followed by NDM, currently representing the greatest proportion of carbapenemases. Further, *K. pneumoniae* has been the most common CPE isolated in South Africa.^3^ Isolates harbouring more than one carbapenemase have also been reported, with a prevalence of approximately 3.5% in CRE bloodstream infections (BSIs) among hospitalized patients surveyed under the Group for Enteric, Respiratory and Meningeal Diseases Surveillance in South Africa (GERMS-SA) of the National Institute for Communicable Diseases.^3^

Reported data on CPEs in the Cape Town metropole have been limited in scope, consisting of institutional reports or outbreak investigations.^8,13^ Thus, despite frequent isolation of suspected CPEs across both the public and private sector, the molecular epidemiology and repertoire of molecular determinants of resistance remains largely unknown. A greater understanding of the CPE resistome will better inform therapeutic decisions with regard to novel antibiotics such as BLICs and repurposed older agents, while also improving antimicrobial stewardship (AMS), by facilitating evidence-based development of empirical and directed therapy protocols. Furthermore, effective infection prevention and control (IPC) interventions are hampered by limited baseline data institutionally and across the metropole. Thus, establishment of prevalent clonal types and mobile genetic elements is critical to the rapid institution of appropriate IPC measures directed at novel outbreaks. From a clinical and pathophysiological point of view, investigation of the virulome is important to guide further clinical and basic science research aimed at reducing morbidity and mortality associated with CREs.

Therefore, this study aimed to evaluate the resistome, virulome, mobilome and molecular epidemiology of the carbapenem-resistant *K. pneumoniae* (CRKP) subset of clinical isolates from hospitalized patients enrolled in a multicentre study of CRE within the Cape Town Metropole, across all sectors.

## Methods

### Clinical isolates

Participants were enrolled in a prospective, multicentre, cross-sectional study between 1 November 2020 and 30 November 2022, from three public-sector hospitals [Groote Schuur Hospital (GSH), Red Cross War Memorial Children’s Hospital (RCWMCH) and Tygerberg Hospital (TBH)] and three private-sector hospitals (Mediclinic Panorama, Netcare Christiaan Barnard Memorial Hospital, Netcare Blaauwberg Hospital) in Cape Town, Western Cape, South Africa. Hospitalized participants with a CRE, based on phenotypic testing, cultured during routine clinical care from any anatomical site (other than rectal swabs and stool) during the study period were eligible for inclusion. Multiple isolates from the same participant and isolates from surveillance cultures were excluded. Additional information on participant enrolment and phenotypic microbiological testing are described in the Supplementary Methods (available as Supplementary data at *JAC-AMR* Online).

Viable CRKP isolates (*n* = 85/86) underwent WGS and are included in this analysis. Non-CRKP isolates, from participants enrolled as described, did not undergo WGS.

### WGS

Viable CRKP isolates (*n* = 85/86) were subcultured overnight on 2% blood agar in an oxygen-enriched incubator at 37°C. Genomic DNA was extracted using the Quick-DNA Fungal/Bacterial Miniprep Kit (Zymo Research Corp., Irvine, CA, USA) followed by library preparation after DNA quality control using the Illumina DNA Prep Kit (Illumina, San Diego, CA, USA) according to the manufacturer’s instructions. Library quality control was performed using an Agilent DNA 1000 kit (Agilent Technologies, CA, USA) and Qubit^™^ dsDNA HS Assay Kit (Invitrogen, Life Technologies, CA, USA). Each sequencing run contained the PhiX V3 internal sequencing control (Illumina) spiked at 2%. Sequencing was performed on an Illumina^®^ MiSeq^™^ instrument using a MiSeq^™^ Reagent Kit v2 (300-cycle) (Illumina) as per the manufacturer’s instructions.

### Bioinformatics

Duplicate reads were removed with SuperDeduper (v1.3.0), and reads trimmed with Trimmomatic (v0.39) and visualized with FastQC (v0.12.1).^14–16^ SPAdes (v3.13.0) was used for *de novo* assembly and assemblies were polished with Pilon (v1.24).^17,18^ All complete *K. pneumoniae* genomes from the NCBI RefSeq database were downloaded and core-genome distances estimated using popPUNK with the *K. pneumoniae* v3 database from the Pathogen Informatics and Modelling group at EMBL-EBI (https://www.bacpop.org/poppunk).^19,20^ Broadly representative candidate reference genomes were selected using a k-means clustering algorithm. Scaffolds generated by SPAdes were compared with the candidate reference genomes using MUMmer (v3.23) and the reference selected with the highest average identity.^21^ The selected reference (GCF_024498655.1) was used for reference-guided scaffolding using Ragtag (v2.1.0) and assemblies evaluated using QUAST (v5.0.2).^22,23^

Antimicrobial resistance genes and resistance-associated mutations were detected using ResFinder (v4.1) with the ResFinder database (24 May 2022) and Kleborate (v2.3.0).^24,25^ Plasmid replicons were detected using PlasmidFinder (v2.1) with the PlasmidFinder database (18 January 2023) from assemblies and plasmids assembled *de novo* using plasmidSPAdes.^26,27^ Contigs with carbapenemase genes detected were evaluated using mlplasmids and RFPlasmid to predict the contig’s chromosomal or plasmid origin.^28,29^ Carbapenemase gene context was visualized using SnapGene software (v7.0.2) (www.snapgene.com) after contig annotation using Prokka (v1.14.6).^30^

Capsule loci were identified using Kaptive.^31,32^ MLST was performed using the MLST 2.0 tool from the Center for Genomic Epidemiology (https://cge.food.dtu.dk/services/MLST).^33^ Virulence genes were detected using MyDbFinder 2.0 (https://cge.food.dtu.dk/services/MyDbFinder) using the VFDB (3 May 2023), Kleborate (v2.3.0) and a curated database of *K. pneumoniae*-specific virulence genes previously described by Jati *et al*.^25,34,35^

Phylogenetic analysis was performed using Parsnp and the tree annotated using the Interactive Tree of Life (iTOL).^36,37^ Assemblies were clustered using popPUNK with the *K. pneumoniae* v3 database from the Pathogen Informatics and Modelling group at EMBL-EBI and visualized using GrapeTree.^20,38^ Bar graphs were generated using GraphPad Prism version 10.0.2 for macOS, GraphPad Software, Boston, MA, USA, (www.graphpad.com).

## Ethics

Ethics approval for the main study (HREC 096/2020), isolate and clinical data biorepository (HREC R009/2020), as well as informed consent and clinical questionnaire were obtained from the University of Cape Town Human Research Ethics Committee. Informed consent was obtained from all participants or their proxies.

## Results

### Molecular epidemiology

**Figure 1.**
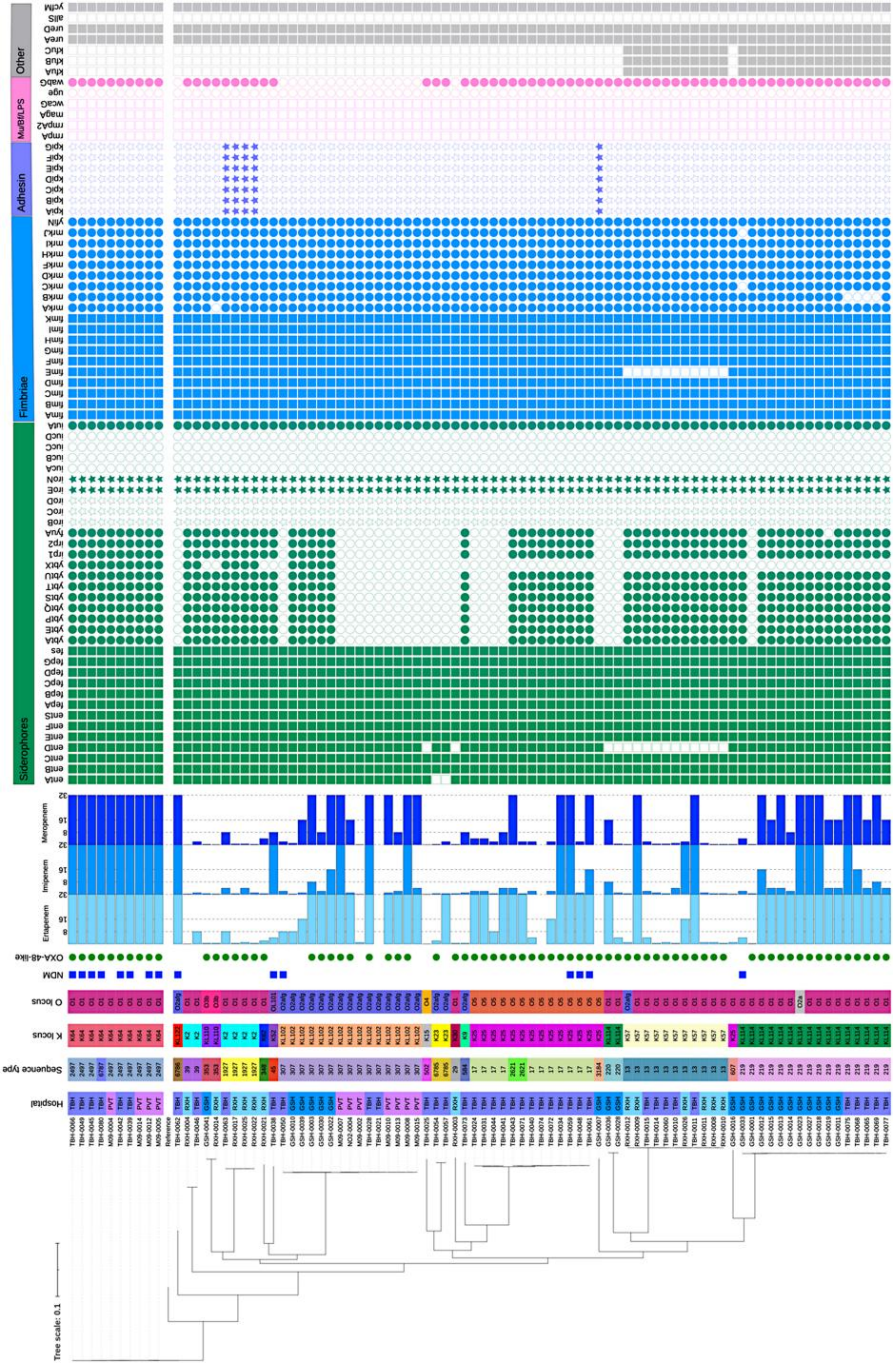
Isolate data. A core-genome SNP phylogenetic tree with all study isolates and the assembly reference GCF_024498655.1 constructed using Parsnp is shown. The tree was visualized and annotated with iTOL. Colour strips indicate the institution of origin, ST, K and O loci. Detected carbapenemase genes are indicated, along with bar graphs showing the MIC (mg/L) of phenotypically tested carbapenems. Detected virulence genes are shown grouped by function. The siderophore gene clusters/loci shown are enterobactin (*entABCDEFS*, *fepABCDG*, *fes*), yersiniabactin (*ybtAEPQSTUX*, *irp1*, *irp2*, *fyuA*), salmochelin (*iroBCDEN*) and aerobactin (*iucABCD*, *iutA*). Relevant to adhesion, genes encoding and regulating fimbriae, (*fimABCDEFGHIK*, *mrkABCDFHIJ*, *yfiN)* and the chaperone-usher pili system *kpiABCDEFG* are shown. Regulator of mucoid phenotype genes (*rmpA* and *rmpA2*), mucoviscosity-associated gene A (*magA*), biofilm-associated *wcaG* gene, LPS biosynthesis *wabG* gene and LPS-regulating uridine diphosphate galactonate 4-epimerase gene (*uge*) are shown. Other genes, clusters or loci shown are the *Klebsiella* ferric iron uptake (*kfuABC*), urease (*ureAD*), allantoin metabolism (*allS*) and non-fimbrial adhesin *ycfM* genes. RXH, Red Cross War Memorial Children’s Hospital; TBH, Tygerberg Hospital; GSH, Groote Schuur Hospital; PVT, Private sector hospital.

**Figure 2.**
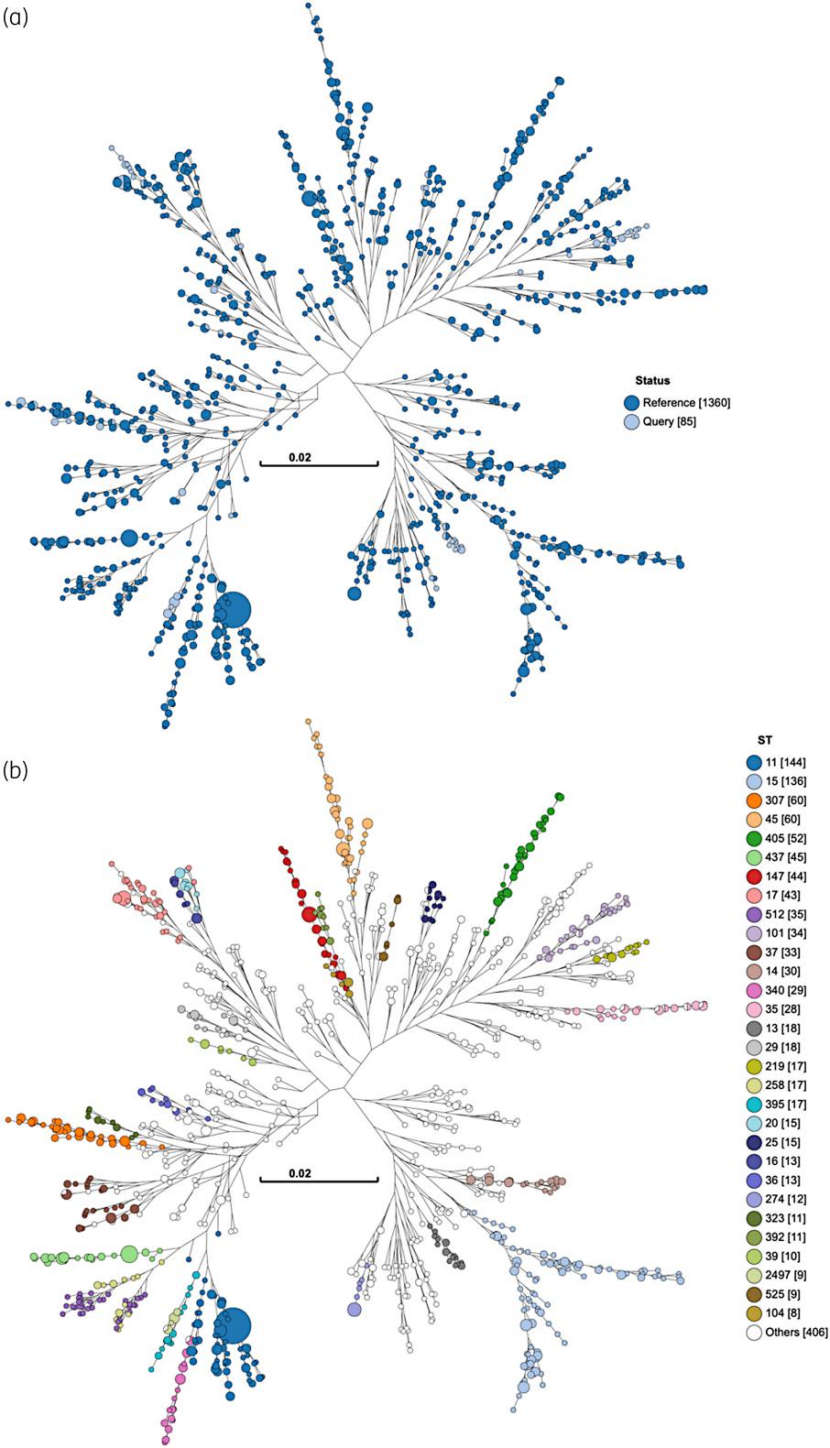
Genome clustering. A neighbour-joining tree constructed from core-genome distances using GrapeTree is shown using the *K. pneumoniae* v3 database from the Pathogen Informatics and Modelling group at EMBL-EBI. (a) Study samples are labelled as ‘Query’ and database entries as ‘Reference’. (b) Nodes are labelled with the ST.

MLST revealed 20 different STs including three novel STs: ST6785 [*n* = 2/85; a single locus variant (SLV) of ST280], ST6786 (*n* = 1/85; a SLV of ST12) and ST6787 (*n* = 1/85; an SLV of ST2497). The most common STs were ST219 at 19% (*n* = 16/85), ST307 at 18% (*n* = 15/85), ST17 at 13% (*n* = 11/85), ST13 at 13% (*n* = 11/85) and ST2497 at 11% (*n* = 9/85), with the remaining STs each having a prevalence of 1%–5%. Significant heterogeneity was noted between hospitals, with ST17 being the most common at TBH, ST219 at GSH, ST13 at RCWMCH and ST307 at sampled private sector hospitals, as shown in Figure 1. Clustering of isolates relative to the *K. pneumoniae* v3 database from the Pathogen Informatics and Modelling group at EMBL-EBI^20^ is shown in Figure 2.

### Resistome

A carbapenemase enzyme-encoding gene was detected in 88% (*n* = 75/85) of isolates. Sixty-four of the 85 isolates harboured a single carbapenemase gene: 71% (*n* = 60/85) were *bla*_OXA-48-like_ (*bla*_OXA-48_ = 38, *bla*_OXA-181 _= 20, *bla*_OXA-232 _= 2) and 5% (*n* = 4/85) were *bla*_NDM-1_. Co-occurrence of two carbapenemase genes was detected in 13% (*n* = 11/85) of the isolates. Eight isolates (all either ST2497 or ST6787) harboured both *bla*_NDM-1_ and *bla*_OXA-232_; and three isolates (all were ST17) harboured both *bla*_NDM-1_ and *bla*_OXA-181_. Detected carbapenemase genes are shown with phenotypic carbapenem susceptibility testing data in Figure 1.

In isolates where a carbapenemase-encoding gene was not detected (12%, *n* = 10/85), truncation of 30%–60% of the *ompK35* gene was detected in five (all were ST307), one of which also had a glycine-aspartate (GD) insertion in the *ompK36* extracellular loop 3 region. A further isolate (ST6785) without a detected carbapenemase-encoding gene had a significant truncation of 82% of *ompK36*. In general, across all isolates with and without a detected carbapenemase gene, co-detection of *ompK35* truncation with *ompK36* GD insertion was found in 21% (*n* = 18/85; all either ST307 or ST219). Truncation of *ompK35* without *ompK36* GD insertion was also detected in all ST2497, ST6787 and remaining, bar one, ST307 isolates.

**Figure 3.**
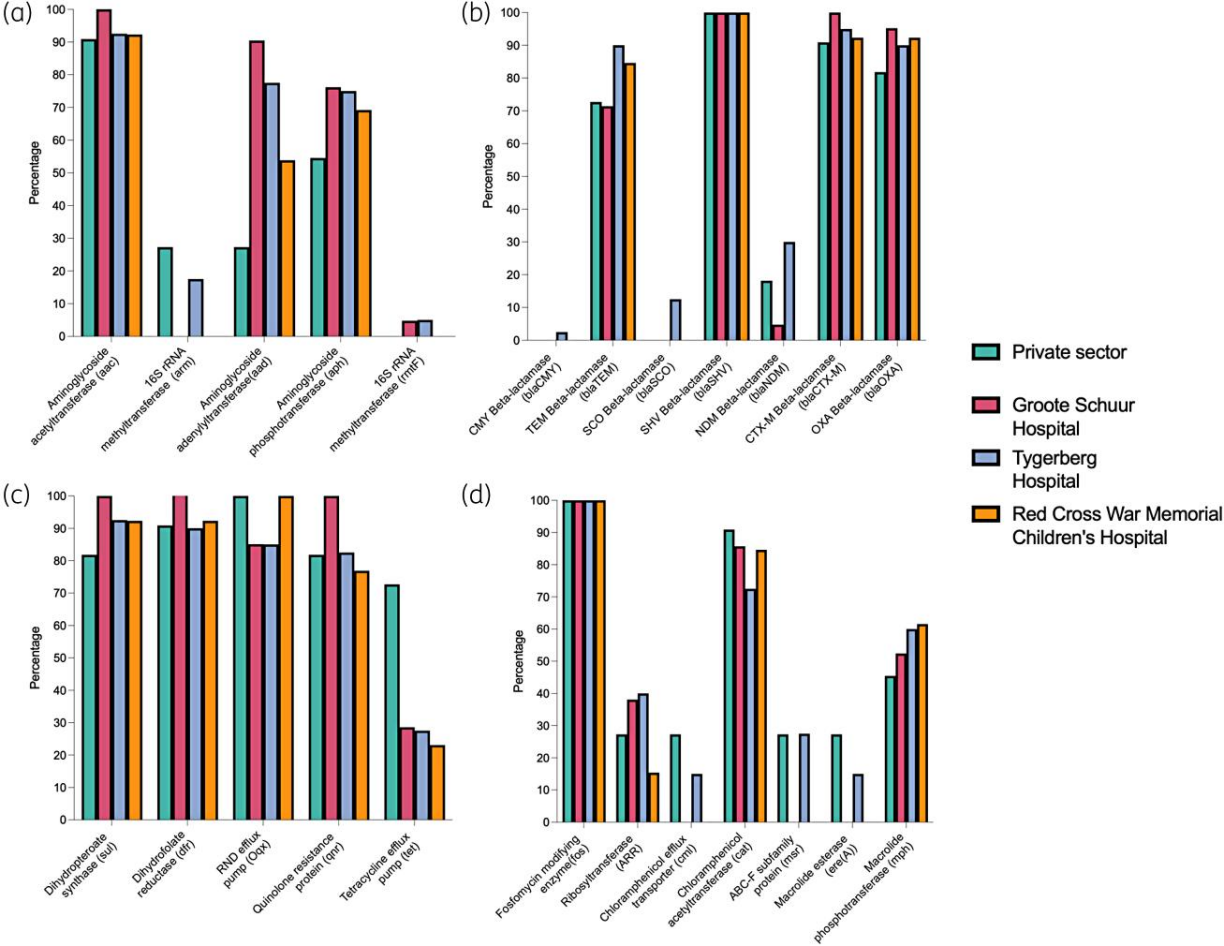
Resistance genes. Bar graphs of institution-specific prevalence of detected resistance genes is shown for (a) aminoglycosides, (b) β-lactams, (c) trimethoprim/sulfamethoxazole, fluoroquinolones and tetracyclines, (d) fosfomycin, rifamycins, macrolides and chloramphenicol.

Regarding other resistance determinants, *bla*_SHV_, *bla*_CTX-M_, *bla*_TEM_, *bla*_SCO_ and *bla*_CMY_ genes were detected in 100% (*n* = 85/85), 95% (*n* = 81/85), 82% (*n* = 70/85), 6% (*n* = 5/85) and 1% (*n* = 1/85) of isolates, respectively. Genes encoding fluoroquinolone resistance, *qnr* and *oqx*, were detected in 86% (*n* = 73/85) and 82% (*n* = 70/85) of isolates, respectively. In 29% (*n* = 25/85) of isolates, both *gyrA* and *parC* mutations were detected, while only *gyrA* mutations were detected in 20% (*n* = 17/85). Aminoglycoside acetyltransferase (*aac*), phosphotransferase (*aph*), adenylyltransferase (*aad*), *arm* 16S rRNA methyltransferase and *rmtF* 16S rRNA methyltransferase genes were detected in 94% (*n* = 80/85), 72% (*n* = 61/85), 71% (*n* = 60/85), 12% (*n* = 10/85) and 4% (*n* = 3/85) of isolates, respectively. The colistin resistance-associated *mcr* gene was not detected in any isolates. Institution-specific prevalence of resistance genes is shown in Figure 3. A complete list of resistance genes detected for each isolate is shown in Table S1.

### Virulome

The enterobactin (*ent*) and yersiniabactin (*ybt*) siderophore virulence gene clusters were detected in 100% (*n* = 85/85) and 73% (62/85) of isolates, respectively. A cluster was considered present when >50% of the genes in the cluster were detected. The *ybt* gene cluster was associated with ICE*Kp5* in 44% (*n* = 27/62), ICE*Kp12* in 27% (*n* = 17/62), ICE*Kp4* in 23% (*n* = 14/62) and ICE*Kp11* in 6% (*n* = 4/62) and the *ybtX* gene was absent in 66% (*n* = 41/62) of isolates. The ferric uptake system *kfuABC* was present in 32% (*n* = 27/85) of isolates, constituting all ST13 and ST219 isolates.

Complete salmochelin (*iro*) and aerobactin (*iuc*) siderophore, genotoxin colibactin (*clb*) and regulator of mucoid phenotype (*rmpADC*) gene clusters were not detected in any isolates. The mucoviscosity-associated gene A (*magA*) and biofilm-associated *wcaG* gene were not detected in any isolates.^39–41^

The type 1 (*fim*) and type 3 (*rmk*) fimbriae gene clusters were complete in 87% (*n* = 74/85) and 93% (*n* = 79/85) of isolates, respectively. The *kpi* gene cluster, previously detected in carbapenem-resistant ST15 *K. pneumoniae* and associated with adherence and biofilm formation, was detected in five isolates.^42^ Four of these isolates were ST1927 and one was ST3184. The *ycfM* gene, which encodes a non-fimbrial adhesin that facilitates adhesion to abiotic surfaces, was detected in all isolates.^43^ The LPS biosynthesis gene associated with colonization capacity, *wabG*, was detected in 80% (*n* = 68/85), while the LPS-regulating uridine diphosphate galactonate 4-epimerase gene (*uge*) was not detected in any isolates.^44,45^ The *ureA* and *ureD* genes from the urease gene cluster were detected in all isolates.

The most common K loci were KL114 at 21% (*n* = 18/85), KL102 at 18% (*n* = 15/85), K25 at 18% (*n* = 15/85), K57 at 13% (*n* = 11/85) and K64 at 12% (*n* = 10/85). The most common O loci were O1 at 54% (*n* = 46/85), O2afg at 24% (*n* = 20/85) and O5 at 16% (*n* = 14/85). Substantial heterogeneity in K locus prevalence between institutions was noted, with KL114 being the most common at GSH (62%, *n* = 13/21), K57 at RCWMCH (46%, *n* = 6/13), K25 at TBH (33%, *n* = 13/40) and KL102 in the private sector (64%, *n* = 7/11).

A curated set of *K. pneumoniae*-specific virulence genes is shown in Figure 1, along with K and O loci. All virulence genes detected using the VFDB database are shown in Table S1.

### Mobilome

**Figure 4.**
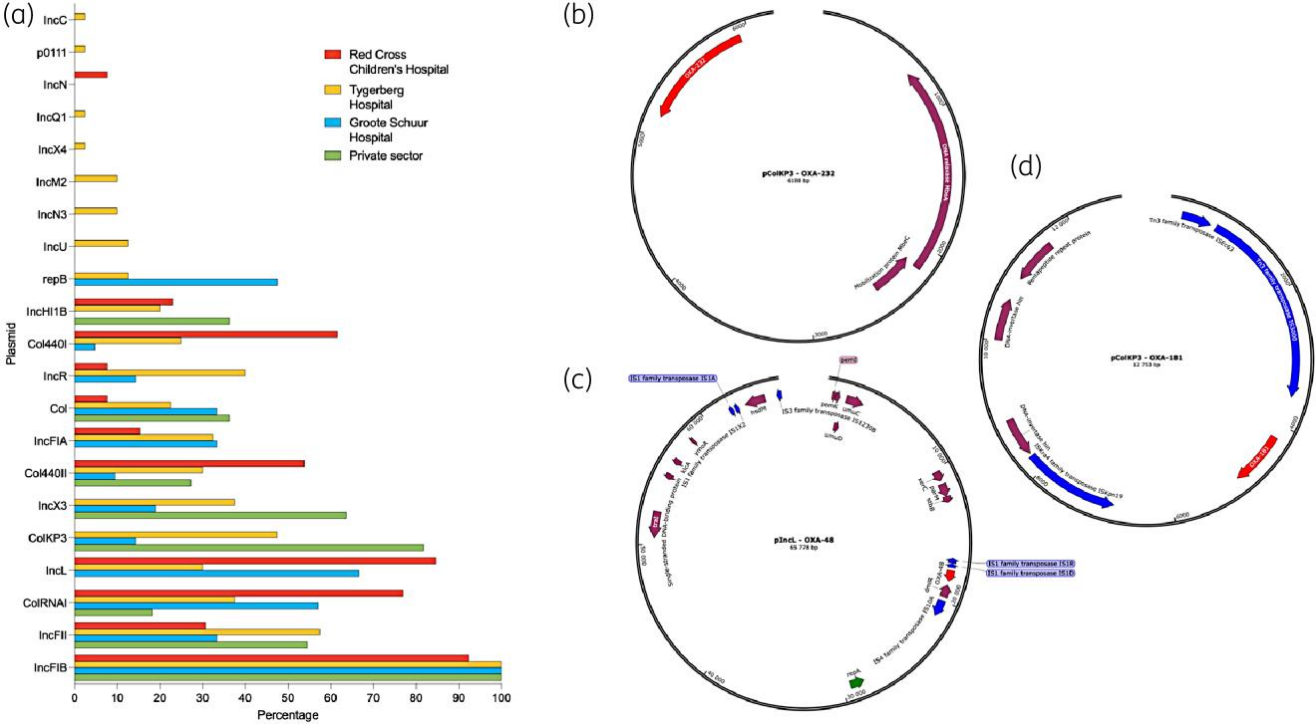
Plasmids. (a) A bar graph of detected plasmid replicon types from assemblies, separated by institution, is shown. The bars indicate the percentage of isolates from each institution in which the plasmid replicon type was detected. The genomic context of carbapenemase genes in contigs generated by plasmidSPAdes and annotated using Prokka is shown for (b) pColKP3 with *bla*_OXA-232_, (c) pIncL with *bla*_OXA-48_, (d) pColKP3 with *bla*_OXA-181_. Hypothetical proteins are not shown.

A total of 21 plasmid replicon types were detected. The most common types were IncFIB, IncFII, ColRNAI, IncL and ColKP3, detected in 99% (*n* = 84/85), 47% (*n* = 40/85), 46% (*n* = 39/85), 44% (*n* = 37/85) and 37% (*n* = 31/85) of isolates, respectively (Figure 4a). A carbapenemase gene was detected on plasmid contigs assembled *de novo* using plasmidSPAdes in 39% (*n* = 33/85) of isolates (Figure 4b–d).^27^ For these assembled contigs, *bla*_OXA-181_ was detected on a contig with a ColKP3 type replicon in 83% (*n* = 19/23) of cases, *bla*_OXA-232_ was detected on a contig with a ColKP3 type replicon in 100% (*n* = 10/10) of cases and *bla*_OXA-48_ was detected on a contig with an IncL type replicon in 5% (*n* = 2/38) of cases. In two isolates, the contig containing the *bla*_OXA-181_ gene contained dual replicon types, IncX3 and ColKP3 in one, and IncFIB(K) and IncC in the other.

For other isolates, carbapenemase genes were present on contigs likely of plasmid origin as determined by mlplasmids^28^ and/or RFplasmid,^29^ but could not be localized to a typeable *de novo* plasmidSPAdes-assembled^27^ sequence. Nonetheless, the IncL replicon was detected in the assemblies of 97% (*n* = 37/38) of isolates with a *bla*_OXA-48_ gene. The IncFIB(pNDM-Mar) and IncHI1B(pNDM-MAR) replicons were detected in all isolate assemblies with both *bla*_OXA-232_ and *bla*_NDM-1_ (ST2497 or ST6787) (in addition to ColKP3 associated with *bla*_OXA-232_). The replicon types IncFIB(K), IncFII(K), IncFIA(HI1), IncM2, IncR and IncX3 were detected in all isolate assemblies with both *bla*_OXA-181_ and *bla*_NDM-1_ (ST17) (in addition to ColKP3 associated with *bla*_OXA-181_). Isolates with only *bla*_NDM-1_ were more heterogeneous (ST307: *n* = 1; ST38: *n* = 1; ST6786: *n* = 1; ST219 *n* = 1). An IncFIB and IncFII replicon type was detected in all four of these isolate assemblies in addition to IncFIA in one, incR in one and IncQ1 and IncM2 in another.

## Discussion

Healthcare-onset infections continue to pose a huge medical burden to public health worldwide. *K. pneumoniae*, one of the WHO priority nosocomial pathogens, causes a broad spectrum of disease and frequently acquires resistance to commonly used antibiotics, particularly in ICUs.^4,46^ Thus, evaluating the molecular epidemiology, resistome, virulome and mobilome of CRKP isolates is pivotal to inform data-driven AMS and IPC interventions.

In this cross-sectional survey across public-sector and private-sector hospitals from Cape Town, CRKP isolates have been characterized. Similar to previous reports from South Africa,^3^  *bla*_OXA-48-like_ carbapenemases were the most common, with *bla*_NDM_ following. Notably, co-detection of two carbapenemase enzymes (*bla*_OXA-48-like_ and *bla*_NDM_) occurred in 13% of isolates (all either ST17, ST2497 or ST6787) and was more common than detection of *bla*_NDM_ alone. Emergence of MBL-producing isolates is a concern as there are no available BLICs in South Africa, at the time of writing, with *in vitro* activity.^47^ Ceftazidime/avibactam, in combination with aztreonam, is currently the preferred regimen for organisms with MBLs, such as NDM, and *in vitro* susceptibility has been shown for isolates with both MBL and serine-β-lactamases.^48,49^ However, both agents have limited availability and potentially prohibitive costs in South Africa while alternative active agents, such as polymyxins and cefiderocol, have similar barriers to use and/or are associated with significant toxicity.^50,51^

All carbapenemase genes were located on contigs linked to plasmids, further highlighting the need for effective IPC. Based on replicon typing of *de novo*-assembled plasmid sequences, all *bla*_OXA-232_ and most *bla*_OXA-181_ genes were co-localized with ColKP3 replicons. ColKP3-type plasmids carrying *bla*_OXA-181_ and *bla*_OXA-232_ have previously been described in a neonatal unit in India (ST15 and ST48) and an emergent ST15 clone in China.^47,52^ The IncL replicon was co-localized with *bla*_OXA-48_ on *de novo*-assembled plasmid sequences and its replicon was detected in isolate assemblies with *bla*_OXA-48_ that could nonetheless not be co-localized with a typeable replicon. IncL-type plasmids have previously been described as the most common plasmid type harbouring *bla*_OXA-48_.^53^

While *bla*_NDM_ genes could not be localized to *de novo*-assembled plasmid sequences, mega plasmids with replicons IncFIB(pNDM-Mar):IncHI1B(pNDM-MAR), found in ST2497 and ST6787 isolates in this study, have previously been shown to encode *bla*_NDM_.^54^ The replicon types IncX3, IncFIB(K), IncFII(K), IncFIA(HI1), IncM2, IncR, IncFIA, IncQ1 and IncM2 detected in other *bla*_NDM_-carrying isolates have all previously been shown to harbour *bla*_NDM_, with IncX3 being the most common.^54^

Although no known carbapenemases were detected in 10 (12%) isolates, 5 had truncations of *ompK35*, which may contribute to carbapenem resistance, and one had a significant truncation of *ompK36*.^55^ In addition to *ompK35* truncation, one of these isolates had an *ompK36* GD insertion, which has been shown to cause pore constriction, resulting in elevated carbapenem MICs.^56^ This highlights the importance of proteome remodelling of the outer membrane in *K. pneumoniae* antibiotic permeability modulation.^57^

Iron acquisition, facilitated by siderophores, is essential for *K. pneumoniae* replication and virulence. Unsurprisingly, the *ent* gene cluster was detected in all isolates. However, lipocalin 2, an innate immune protein produced by neutrophils and mucosal surfaces, specifically binds enterobactin and disrupts iron acquisition.^58^ Lipocalin 2 cannot bind to the siderophore yersiniabactin and thus yersiniabactin has been shown to promote respiratory tract infections.^58^ In this study, the *ybt* gene cluster was detected in approximately three-quarters of the isolates. Deletion of *ybt*X in two-thirds of isolates is interesting as *ybt*X deletion has previously been shown to be associated with reduced inflammation during lung infection with *K. pneumoniae*.^59^ Further, related to iron uptake, the *kfu*ABC system was detected in one-third of isolates (ST13 and ST219) and has been associated with a greater tissue-invasive potential and metastatic infection.^60^

Analysis revealed several STs with significant heterogeneity between institutions. Specifically, different STs predominated in each of the respective institutions, suggesting local in-hospital circulation. The ST307 and ST17 clones, predominant in the private sector and TBH, respectively, have both been reported globally as important antimicrobial drug-resistant clones.^7,61,62^ The ST13 clone, predominant at RCWMCH, has previously been described as an emergent clone associated with carbapenem resistance in Europe.^63^

Only six isolates had a capsular locus (K2) that is associated with hypervirulence, and none of these isolates had the complete *iro*, *iuc* or *rmp*ADC virulence gene clusters associated with the hypervirulence genotype.^64^ There are limited published data available on the prevalence of hypervirulent *K. pneumoniae* in South Africa, thus the lack of convergence of carbapenem resistance and hypervirulence, as has previously been described in China, is of uncertain significance.^65^ The hypervirulence-associated *magA* gene was also not detected in any isolates. In general, substantial heterogeneity in K locus prevalence, correlating with dominant ST, was noted between institutions with smaller diversity in O type.

This study had several limitations. Long-read sequencing was not performed, which complicated the assembly of complete plasmid sequences.^35^ Thus, carbapenemase gene-containing plasmidSPAdes-assembled contigs could only be replicon typed in 44% of isolates. As a result, limited inferences could be made regarding *bla*_NDM_ and *bla*_OXA-48_ carbapenemase gene context, beyond detection of replicon types commonly associated with these genes in the isolate assemblies in general. A major barrier to participant recruitment was SARS-CoV-2-related policy such as changes in IPC practice. Fewer hospitals participated and participant recruitment was heavily impacted by policies such as increased approval process stringency and limitation of study-related staff movement within participating institutions. Additionally, travel within and outside South Africa, as well as largely temporary changes in community and hospital IPC practices, may have influenced CRKP transmission dynamics, limiting the applicability of data to current CRKP epidemiology. This siloed practice may have contributed to the study finding of institutionally dominant, rather than regional, clones.

In conclusion, this study evaluated CRKP epidemiology in Cape Town and found that the most prevalent carbapenemase gene was *bla*_OXA-48-like_, in keeping with previously described CRKP epidemiology in South Africa.^3^ Hypervirulent CRKP isolates were not detected in this collection. Emerging clones that may harbour both *bla*_OXA-48-like_ and *bla*_NDM_, such as ST17, ST2497 and the novel ST6787, are a concern due to the limited availability of appropriate antimicrobial agents, particularly in the South African public sector. Regarding molecular epidemiology, institutionally dominant, rather than regional clones, were found.

## Supplementary Material

dlae050_Supplementary_Data

## Data Availability

The data that supports the findings of this study are available in the Supplementary data of this article and uploaded to the NCBI with the primary accession PRJNA1035075. BioSample accessions are indicated in Table S1.

## Members (collaborators) of the Cape Town Antimicrobial Stewardship Study Alliance (CT-ASSA)

Mediclinic Hospital group: Claudine Page, Elizabeth Schoeman, Elizma de Klerk, Karin Lategan, Karlien Pienaar, Liezl Henning, Mandy Du Plessis, Nomfundo Maseko, Salome Nel, Melenie Narainsamy, Michelle Vermeulen, Narissa du Toit, Teresa van Heerden. Netcare Hospital Group: Liza Sitharam, Asa Barendse, Dane Nagel, Jacqueline Prince, Letitia Vass, Rileen Strauss, Rushana Fakier. Pathcare Laboratory group: Catherine Samuel, Marelieze van Zyl, Leigh-Ann Isaacs, Shareefa Hendricks, Amy Dodd, Reecka Daniels. University of Cape Town Medical Microbiology Group: Widaad Zemanay, Judi Van Heerden, Nchimunya Hapeela, Parveen Brown, Zubayr Daniels. Life Healthcare Hospital Group: Sharon Vasuthevan, Enid Scott, Esmeralda Ricks, Patricia Curle. Lancet Laboratory Group: Justyna Wojno.

## Supplementary data

Supplementary Methods and Table S1 are available as Supplementary data at *JAC-AMR* Online.
