# Supplementary material for: Carbapenem-resistant Klebsiella pneumoniae among hospitalized patients in Cape Town, South Africa: molecular epidemiology and characterization
Source: JAC Antimicrob Resist. 2024 Mar 25;6(2):dlae050. doi: 10.1093/jacamr/dlae050 (PMC10963078; doi:10.1093/jacamr/dlae050)
Supplement: dlae050_Supplementary_Data [file dlae050_supplementary_data.zip › JAC-AMR-2023-259.R2 Supplementary Methods.docx]

**Supplementary methods**

**Participant enrolment**

| Inclusion criteria | - Participant hospitalised at the time of microbiological sampling during routine clinical care - Culture of an isolate from any anatomical site with demonstrated phenotypic non-susceptibility to a carbapenem during routine clinical laboratory testing - Isolate identified as a member of the Enterobacterales order during routine clinical laboratory testing |
| --- | --- |
| Exclusion criteria | - Duplicate isolates from the same sampling event - Prior inclusion of the participant in this study - Isolates derived from a surveillance culture (including rectal swabs to screen for colonisation) - Isolates with intrinsic reduced susceptibility to imipenem with isolated imipenem non-susceptibility (*Proteus* spp., *Providencia* spp., and *Morganella* spp.) |

**Routine clinical care: Antimicrobial stewardship and infection prevention and control**

The public sector hospitals involved have multiple infectious disease specialist-driven antimicrobial stewardship (AMS) and infection prevention and control interventions whilst the private hospitals have primarily pharmacist-driven AMS programs supported by clinical microbiologists. Both sectors do prospective audit and feedback, antibiotic utilization surveys, peri-operative antibiotic prophylaxis interventions, and have detailed antibiotic guidelines.

**Routine clinical care: Culture and antimicrobial susceptibility testing**

Routine bacterial isolate identification and susceptibility testing was performed at clinical laboratories accredited by the South African National Accreditation System. Isolate identification was performed using the VITEK 2 instrument (bioMérieux, France), or the MALDI-TOF VITEK MS (bioMérieux, France) instrument based on laboratory standard operating procedures (SOP). Susceptibility testing was performed using the VITEK 2 instrument, ETEST (bioMérieux, France) gradient diffusion, Kirby-Bauer disc diffusion or broth micro-dilution as appropriate for the relevant isolate based on the specific laboratory SOP. Antimicrobial breakpoints published by the Clinical Laboratory and Standards Institute (CLSI) or the European Committee on Antimicrobial Susceptibility Testing (EUCAST) guidelines for the relevant year were used for interpretation of susceptibility testing results.
